# Impacts of neonicotinoid seed treatments on soil-dwelling pest populations and agronomic parameters in corn and soybean in Quebec (Canada)

**DOI:** 10.1371/journal.pone.0229136

**Published:** 2020-02-26

**Authors:** Geneviève Labrie, Annie-Ève Gagnon, Anne Vanasse, Alexis Latraverse, Gilles Tremblay

**Affiliations:** 1 Centre de recherche sur les grains Inc. (CÉROM), St-Mathieu-de-Beloeil, Québec, Canada; 2 Département de phytologie, Université Laval, Québec, Québec, Canada; Institut Sophia Agrobiotech, FRANCE

## Abstract

Agricultural soil pests, including wireworms (Coleoptera: Elateridae), are managed primarily with pesticides applied directly to seeds before sowing. Seeds coated with neonicotinoids have been used widely in Quebec (Canada) for several years. To assess the agronomic and economic value of neonicotinoid seed treatments in soybeans and corn in Quebec, trials were conducted from 2012 to 2016 in 84 fields across seven regions in Quebec. We evaluated the effect of neonicotinoid seed treatments on soil pest densities, crop damage and yield. The results showed that 92.6% of corn fields and 69.0% of soybean fields had less than 1 wireworm per bait trap. However, no significant differences in plant stand or yield were observed between treated and untreated corn or soybeans during the study. This study shows that neonicotinoid seed treatments in field crops in Quebec are useful in less than 5% of cases, given the very low level of pest-associated pressure and damage, and that they should not be used prophylactically. Integrated pest management (IPM) strategies need to be developed for soil insect pests to offer effective alternative solutions to producers.

## Introduction

Since the middle of the 1990s, neonicotinoids (i.e. imidacloprid, clothianidin and thiamethoxam) have become the main class of insecticides routinely used to protect seeds and seedlings against injuries caused by soil insects [1, 2, 3]. Corn, canola, soybeans, wheat and cotton are the principal crops grown worldwide for which seed treatments are used on a large scale, with a rapid increase in the acreages treated [1, 4]. A vast body of scientific literature has demonstrated that the scale of use of those insecticides has resulted in widespread contamination of agricultural soils, freshwater resources, wetlands, and non-target vegetation, along with repeated and chronic exposure of the organisms inhabiting these habitats to potentially harmful concentrations of these pesticides [2, 5, 6, 7, 8, 9, 10, 11, 12, 13]. In Canada, widespread contamination of water [14, 15, 16, 17, 18, 19, 20, 21] and impacts on non-target organisms, such as pollinators [22, 23, 24, 25], have already been demonstrated.

In the province of Quebec, Canada, seeds coated with neonicotinoid insecticides are widely used as a prophylactic treatment on almost 100% of corn acreages and canola, and about 60% of soybean acreages, representing over 500,000 ha sown with treated seeds each year [26]. These seed treatments are mainly used to control soil pests, such as white grubs (Coleoptera: Scarabaeidae), seedcorn maggot (Diptera: Anthomyiidae) and wireworms (Coleoptera: Elateridae), and represent an attractive option as “insurance” against those pests because of their relatively low cost, ease of handling and low toxicity to mammals [1, 3, 27, 28]. The treatments are generally used in the absence of any documented increase in pest threats [1, 2, 29], partially driven by current market efficiencies [30], and few studies have examined their usefulness against soil-dwelling insect pests such as wireworms and seedcorn maggots.

Wireworms are considered major soil pests worldwide [31]. More than 1,000 species are found in North America [32] and 370 species in Canada [33, 34, 35], including 30 economically important species. A recent study reported that 9 genera of wireworms occur in Quebec, with the abbreviated wireworm, *Hypnoidus abbreviatus* Say, being the most abundant species, accounting for 72% of all wireworms collected in field crops [36]. Wireworms are early-season pests that can injure seeds and seedlings in spring, reducing the establishment and growth of young plants in the field [37, 38]. Seedcorn maggots (*Delia platura* Meigen, *D*. *florilega* [Zetterstedt, 1845]) are pests of numerous vegetable and field crops and can cause serious economic losses when larvae penetrate the germinating seeds or seedlings and mine cotyledons, small shoots and/or young roots before sprouting occurs [39, 40, 41, 42]. They are usually sporadic pests in field crops, mainly attracted by organic matter or cover crops incorporated into the soil before sowing [43, 44, 45]. In Quebec, the seedcorn maggot is observed sporadically in soybean or corn fields, but its impact has not been evaluated on a large scale.

The debate surrounding the prophylactic use of neonicotinoids has mainly focused on the potential yield increase in corn and soybeans. Recent studies have sought to analyze whether yield differences can be observed in soybean with or without neonicotinoid seed treatments targeting soybean aphids, *Aphis glycines* Matsumura [46, 47, 48, 49, 50, 51, 52, 53, 54, 55, 56]. A few studies demonstrated yield increases with the use of neonicotinoid treated seeds, mainly when more than one type of pest was present in the field [51, 52], while other studies presented no differences. However, those studies focused only on above-ground pests, and none evaluated the usefulness of neonicotinoids against soil-dwelling insect pests in soybean.

In corn, some studies have examined yield differences between treated and untreated seeds [30, 53, 57, 58, 59, 60, 61]; however, the results are inconsistent, with a recent meta-analysis covering 15 years of high dose of neonicotinoids applied to control western and northern corn rootworm in Indiana demonstrating no yield differences [30], while another study spanning 14 years and 91 trials in the southern part of the USA showed a 700 kg/ha higher yield in treated corn [59]. In the northeastern part of Canada, which has different climatic conditions and agronomic practices, no studies have been done to evaluate soil-borne insect pest pressure or the impact of using insecticide seed treatments in corn and soybean.

Our study was designed to evaluate agronomic parameters related to soybean and corn planted with neonicotinoid treated seed or untreated seed on a large scale over a five-year period in the province of Quebec, Canada. The main objectives of this project were to evaluate the impact of neonicotinoid seed treatments on 1) the incidence and abundance of wireworms and other soil-dwelling insect pests and 2) soybean and corn yield.

## Materials and methods

### Experimental sites

In 2012–13, an experiment was conducted at 12 and 13 corn growing sites in the Monteregie region (the main corn growing area), while in 2014–15, the study was conducted at 19 and 24 sites in seven corn producing regions across the province of Quebec, for a total of 68 sites. All sites were on commercial growers’ farms. No specific permissions have been requested to collect insects and agronomic parameters on grower’s farm for the 84 trials of the project. Each site was the property of a grower, and all of them give us the right to collect insects and soil samples. The study did not involve any endangered or protected species. In 2015–16, a study on soybean was conducted at 7 and 9 sites located in the Monteregie and Centre-du-Quebec areas. The locations were carefully chosen to represent sites with high risk factors (sandy soil, grassland as previous crop, no till, organic fertilization; high organic matter) or low risk factors (clay, soybean rotation, conventional tillage, mineral fertilization) to ensure that soil-dwelling pests would be present [31, 40], and to represent the variability observed in field crops in the province. The characteristics of each site are summarized in S1 Table (Appendix).

Each site was sown with two alternating strips of treated seed and untreated seed, repeated three times for a total of 6 plots (plots were 6 to 9 m wide × 200–300 m long). Seeds were sown at a depth of approximately 4.5 cm and with 76-cm spacing between rows. The approximate sowing rate was between 80,000 and 89,000 seeds per hectare (ha) for corn, and 400,000 and 500,000 seeds per ha for soybean. All sites were sown in the direction of the slope to prevent insecticide contamination of untreated plots caused by surface runoff. Fertilization differed according to the growers’ practices and consisted of mineral or organic manure (S1 Table, Appendix).

### Seed treatments

For the corn study, different hybrids as well as different insecticide and fungicide seed treatments were used, depending on the year and the corn heat units (CHU) associated with the region. In 2012, the hybrid used was K293 RR (Horizon Seeds Canada Inc.) treated with Poncho® 600 (insecticide; clothianidin 0.25 mg/seed, Bayer CropScience Inc.) and Maxim® Quattro (fungicides; fludioxonil + metalaxyl-M and S-isomer + azoxystrobin + thiabendazole, Syngenta Canada Inc.). In 2013, the hybrid was HZ872BtGT (Horizon Seeds Canada Inc.) treated with Cruiser Maxx® (insecticide; thiamethoxam 0.25 mg/seed, Syngenta Canada Inc.) and Maxim® Quattro. Hybrids with 2850 CHU were used in both years. In 2014–15, hybrids with different CHU were used depending on the region (R E50G22: RR2, Genuity, 2400 CHU [14 sites]; E61P12 R: RR, Genuity VT Double Pro, 2700 CHU [17 sites] and R E65F12: RR Genuity VT Double Pro 2850 CHU [12 sites], all from Elite®). The seeds were treated with Poncho® 600 and Maxim® Quattro. The seeds used for the control plots in the corn assays between 2012 and 2015 were treated only with Maxim® Quattro (fungicides). Treated and control seeds came from the same seed lots in 2012, 2013 and 2015.

In both years of the soybean study, the plots were sown with the cultivar Montero RR (Prograin) treated with Cruiser Maxx® (Syngenta Canada Inc.), which includes an insecticide (thiamethoxam 0.25 mg/seed) and fungicides (difenoconaxole + metalaxyl-M + sedaxane). The seeds used for the control plots in the soybean assays were treated only with fungicides (difenoconaxole, metalaxyl-M and sedaxane). Treated and control seeds came from the same seed lots.

### Insect sampling

Three 3 × 3 m sampling stations, each covering four rows of crop, were installed in each strip, for a total of 18 stations per site. Stations in the same strip were installed at least 50 m apart. Each site was visited five to eight times during the growing season to install and replace insect traps, collect data on seedling damage, and harvest plants.

Soil insects were sampled using two different methods: bait traps (2012–15 in corn and 2015–16 in soybean) and soil sampling (2014–15 in corn and 2015–16 in soybean). Bait traps were used to sample wireworms, while soil sampling was used to capture wireworms, white grubs and other soil-dwelling pests. One bait trap per station was installed (18 per field) just after sowing (in May). The traps consisted of a 15 × 15 × 15 cm hole in the ground filled with bait (one cup of an equal parts mixture of wheat flour, untreated wheat seeds and oatmeal), and covered with soil. The baits were dug out and destructively inspected in the field once a week. The wireworms in each trap were collected in vials (to be counted and identified in the laboratory), and a new trap was set up near the old one. Five soil samples (10 cm diameter × 15 cm depth) were taken weekly from each strip (i.e. one per sampling station and one between each station, for a total of 30 samples per site). Insects were extracted in the laboratory using Berlese funnels (kept for 24 h under 60W incandescent light bulb) and counted. Identification was done by morphological analysis using a species key [34, 62, 63, 64, 65]. Voucher specimens have been added to the Collection nationale des insectes du Québec (Québec, QC, Canada).

### Plant and seedlings observations

#### Plant stand and seedling damage

Corn and soybean populations were evaluated when the plants were between the 4- to 8-leaf stages. An assessment of plant stand was done by counting all the plants along the two central rows of each station over a length of 3 m.

Once a year, between 2013 and 2016, corn and soybean seedlings (2–6 leaves) were observed at each site to evaluate the main causes of damage. Three seedlings presenting symptoms (less vigorous, small or yellowing or stunted plants) or damage (holes, chewed parts) or dead seedlings were dug out at each station (i.e. 54 seedlings per site). Special care was taken when recovering the seeds to identify holes or galleries made by wireworms or other insects. Seedlings were taken to the laboratory to identify the damage. Any wireworms found were identified to species level.

### Yields

To estimate crop yields under neonicotinoid treated and untreated seed treatments, soybean and corn were mechanically harvested in each strip. For the corn assays, a commercial harvester was used, and the grain car was weighed separately for each strip. For the soybean assays, the two middle rows of each strip were harvested with an experimental plot combine harvester (Wintersteiger AG, Ried im Innkreis, Austria). Subsamples of grain (corn and soybean) were collected to measure moisture in the laboratory and results were reported based on 15% moisture content for corn and 13% for soybean. In 2015, one soybean site (Roxton Pond) was not harvested because of water accumulation during summer.

### Statistical analyses

The corn and soybean data were analyzed separately using a mixed model approach to account for the non-independent spatial associations (pseudoreplication) in the data and the unbalanced replication due to missing data at some of our sites [66, 67]. Plant stands and yields were analyzed using linear mixed models (LMM) assuming a Gaussian distribution of the error and the identity link function. Wireworm captures (bait traps and soil samples) were analyzed using generalized linear mixed models (GLMM) with a Poisson distribution of the error and a log link function. The Poisson distribution is recommended for the analysis of count data which are discrete and positive. Although plant stand is a count variable, it was normally distributed with a constant standard deviation. Proportions of damaged seedling were analyzed using GLMMs with a binomial distribution of the error and a logit link function. This distribution is recommended to analyze proportional data based on number of “success” over a known number of “trials”. The bait traps and soil samples analyses were conducted on the most abundant trapping survey. Because of the high number of zeros, wireworm captures were pooled per treatment per site prior to analysis and the number of traps or soil samples was used as an offset to account for the sampling effort. Only sites with more than five captures of wireworms were included in the models.

All response variables were modelled with seed treatments (treated or untreated) and year as fixed effects. The full model for the plant stands analysis included a random intercept and slope for Site and Block (nested within Site) as random effect, while the bait traps, soil samples and yield analyses, included a random slope for Site. The best random structure for each analysis was then determined using Akaike’s Information Criterion for smaller sample sizes (AICc) [68] using restricted maximum likelihood estimation (REML). Once determined the best random structures, the resulting models were refitted with maximum likelihood (ML) and the seed treatment effect was tested using likelihood ratio tests (LRT). The parameter estimates presented for the final models were evaluated based on restricted maximum likelihood.

Data exploration was carried out following the protocol described in Zuur et al. [69] and the assumptions of the models were checked visually. Analyses were performed using R language [70]. Linear mixed models and generalized linear mixed models were fit with the functions lmer and glmer, respectively, from the package lme4 [71]. The AICc values were calculated using function AICc from the package MuMIn [72] and the LRT test were performed using function drop1.

## Results

### Effect of neonicotinoids on insect populations

#### Corn pests

**Fig 1:**
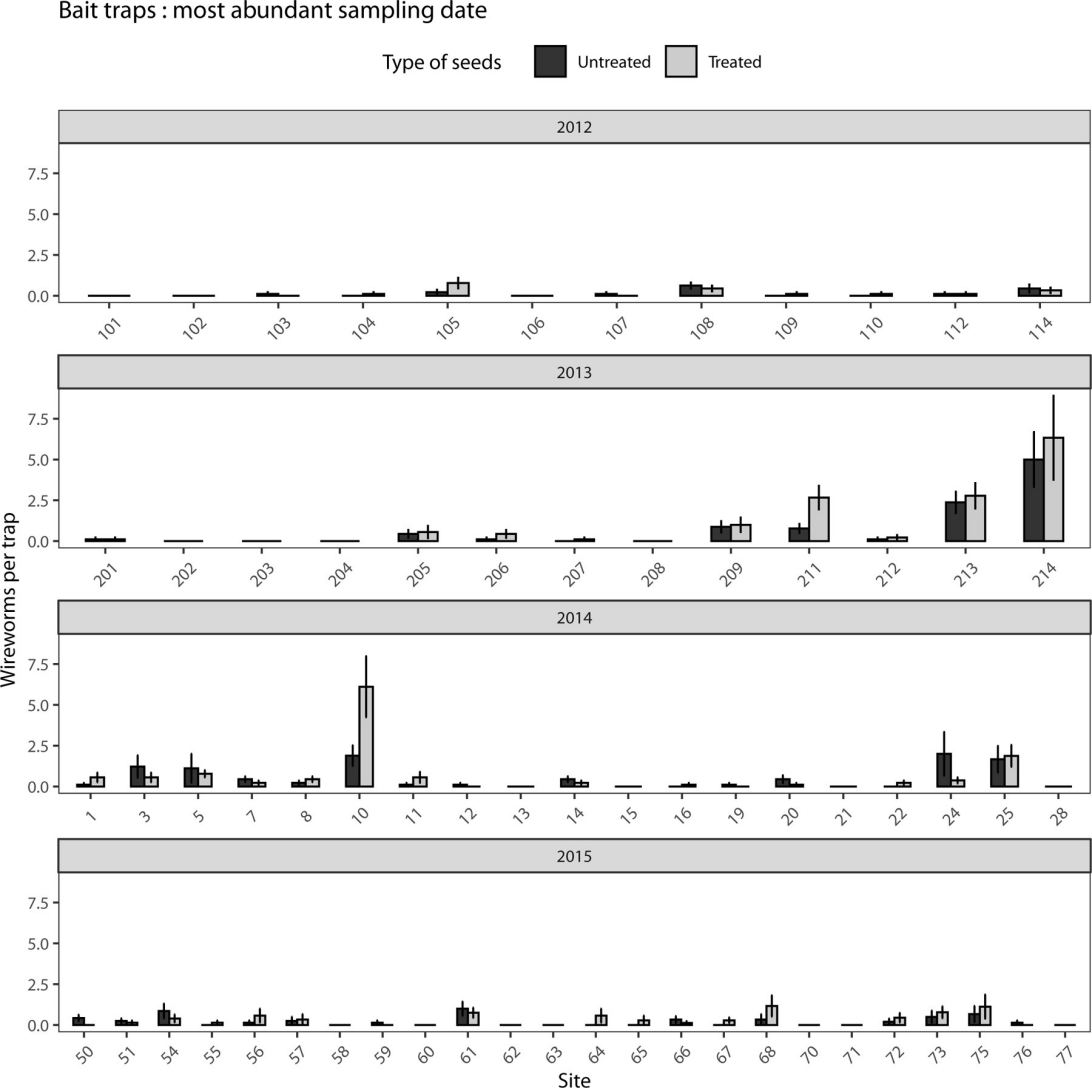
Mean abundance (±SE) of wireworms per bait trap in corn fields with or without neonicotinoid seed treatments (68 sites). The average number of wireworms found at each site for the most abundant visit is presented for each year: (A) 2012, (B) 2013, (C) 2014, (D) 2015. Neonicotinoid seed treatment varies between years; complete information on treatments is described in the Materials and Methods section.

A total of 1,032 wireworms were captured in the bait traps over the four years of the project (2012 to 2015). The mean number of wireworms in each trap per visit varied between 1 and 21. Among the 68 sites sampled, only five exceeded 1 wireworm per trap (three in 2013 and two in 2014; Fig 1). The main species was *H*. *abbreviatus*, representing 56%, 82%, 48% and 76% of the assemblage in 2012, 2013, 2014 and 2015, respectively, followed by *Agriotes mancus* Say and *Melanotus similis* (Kirby, 1837), accounting for 2 to 17% of the assemblage depending on the year. Other wireworms captured belonged to the genera *Limonius*, *Dalopius* and *Ampedus*.

The number of wireworms per bait trap did not differ significantly across locations-years between neonicotinoid treated and untreated strips (LRT; χ_(1)_ = 0.975; *p* = 0.32).

**Fig 2:**
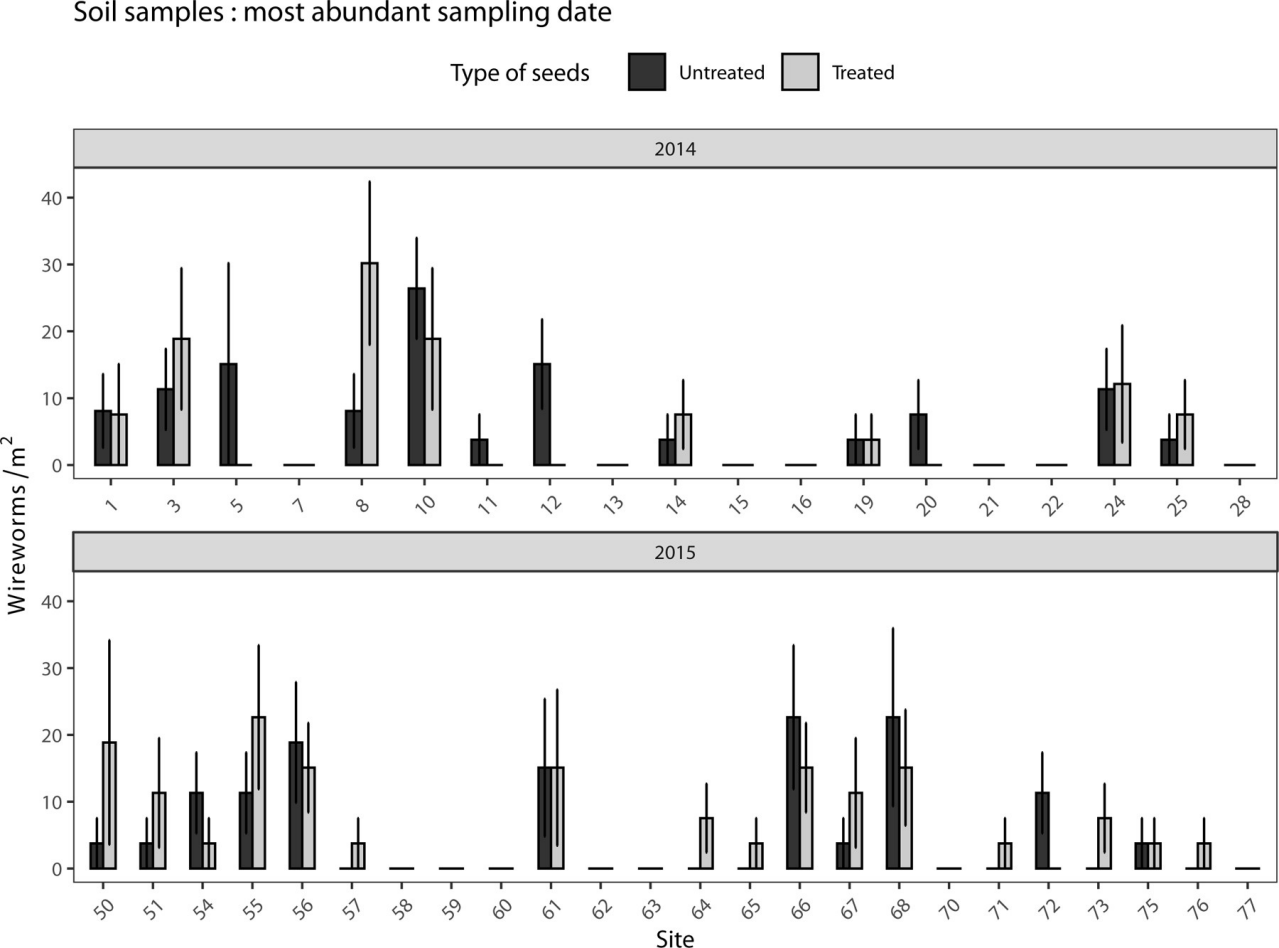
Mean abundance (± SE) of wireworms per m^2^ in soil samples from corn fields with neonicotinoid treated strips and untreated strips in 2014 (A) and 2015 (B) (total of 43 sites).

In 2014 and 2015, the number of wireworms captured in soil samples per sampling period varied between 0 and 94 larvae/m^2^ (Fig 2). The main species observed was *H*. *abbreviatus*, representing 47% and 87% of the assemblage in 2014 and 2015, respectively. For the bait traps, the number of wireworms did not differ significantly between neonicotinoid treated and untreated strips (χ^2^ = 0.728; df = 1; *p* = 0.393; Fig 2).

Other soil insect pests have been observed in bait traps and soil samples, such as seedcorn maggot (*Delia platura* Meigen) pupae, white grubs (*Phyllophaga anxia* [Leconte]), *Ateanius* sp., *Aphodius* sp.), Noctuidae larvae (Lepidoptera), such as black cutworm (*Agrotis ipsilon* [Hufnagel]) and Tipulidae larvae (Diptera). Seedcorn maggots were omnipresent in all fields but did not affect seedlings in a systematic manner (see next section for a description of damage). Of the 239 larvae captured, only 16 were white grubs (*P*. *anxia*); all others belonged to the genera *Ateanius* and *Aphodius*. A total of 84 Tipulidae larvae were observed, of which 54 belonged to the species *Tipula paludosa* (Meigen). These low numbers precluded a comparison of neonicotinoid treated and untreated plots with respect to the abundance of other soil-dwelling insect pests.

#### Soybean pests

**Fig 3:**
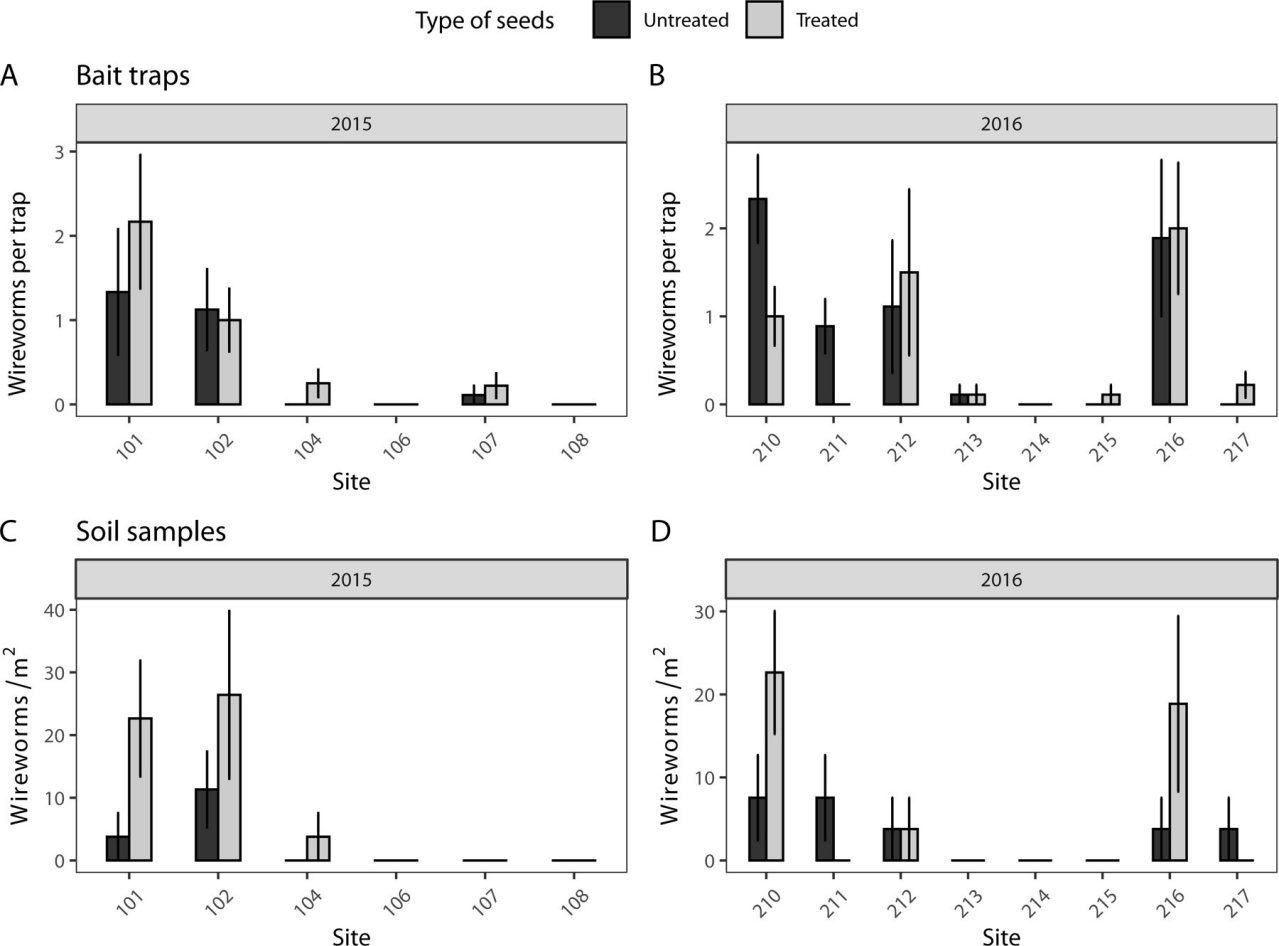
Mean abundance (±SE) of wireworms per bait trap (A, B) and soil sample (C, D) in 2015 and 2016 in soybean fields with or without neonicotinoid seed treatments. in 2015, only 6 of the 7 planted sites were harvested.

A total of 271 wireworms were captured in bait traps over the two years during which this sampling method was used (48 in 2015 and 105 in 2016). The mean number of wireworms captured in bait traps per sampling period varied between 0 and 2.78 (Fig 3A and 3B). The main species observed was *H*. *abbreviatus*, with a total of 241 specimens captured, accounting for 84% and 86% of the assemblage in 2015 and 2016, respectively. The other wireworms collected belonged to the species *A*. *mancus* and the genera *Hemicrepidius*, *Melanotus*, *Limonius* and *Oestodes*. Among the 15 sites sampled, 5 sites (two in 2015 and three in 2016) exceeded a mean of 1 wireworm per trap (Fig 3A and 3B). Wireworm population density did not differ significantly between the neonicotinoid treatment and the control in 2015 (LRT; χ^2^ = 0.407; df = 1; *p* = 0.52) and 2016 (LRT; χ^2^ = 0.044; df = 1; *p* = 0.83).

A total of 72 wireworms were captured in soil samples. The mean abundance varied between 0 and 59/m^2^ or 51 larvae/m^2^ in 2015 and 2016, respectively (Fig 3C and 3D). The main species observed was *H*. *abbreviatus*, with a total of 59 specimens captured, representing 81% of the species during both years of the study. The other wireworms collected belonged to the species *A*. *mancus*, *M*. *similis* and the genera *Hemicrepidius* and *Oestodes*. Only two larvae of *P*. *anxia* were captured on one site in 2015 and five larvae on two sites in 2016. A higher abundance of wireworms was observed in neonicotinoid treatment strips compared to control strips in 2015 (LRT; χ^2^ = 5.21; df = 1; *p* = 0.02) but not in 2016 (LRT; χ^2^ = 0; df = 1; *p* = 0.99).

### Plant stand

Corn plant stands varied between 4 and 7 plants/m during the four-year study. Overall, for all sites and years, no significant differences in corn stand was observed between treated (5.80 ± 0.07 plants/m) and untreated plots (5.73 ± 0.07 plants/m) (LRT; *F*_1, 67.1_ = 3.20; *p* = 0.078).

In soybean, plant stands were very variables among sites (between 16 plants/m and 76 plants/m) during the two-year study. For all sites, no significant differences in the stand of soybean were observed between treated and untreated plots (LRT; *F*_1, 12.14_ = 2.76; *p* = 0.122).

### Seedling damage

**Fig 4:**
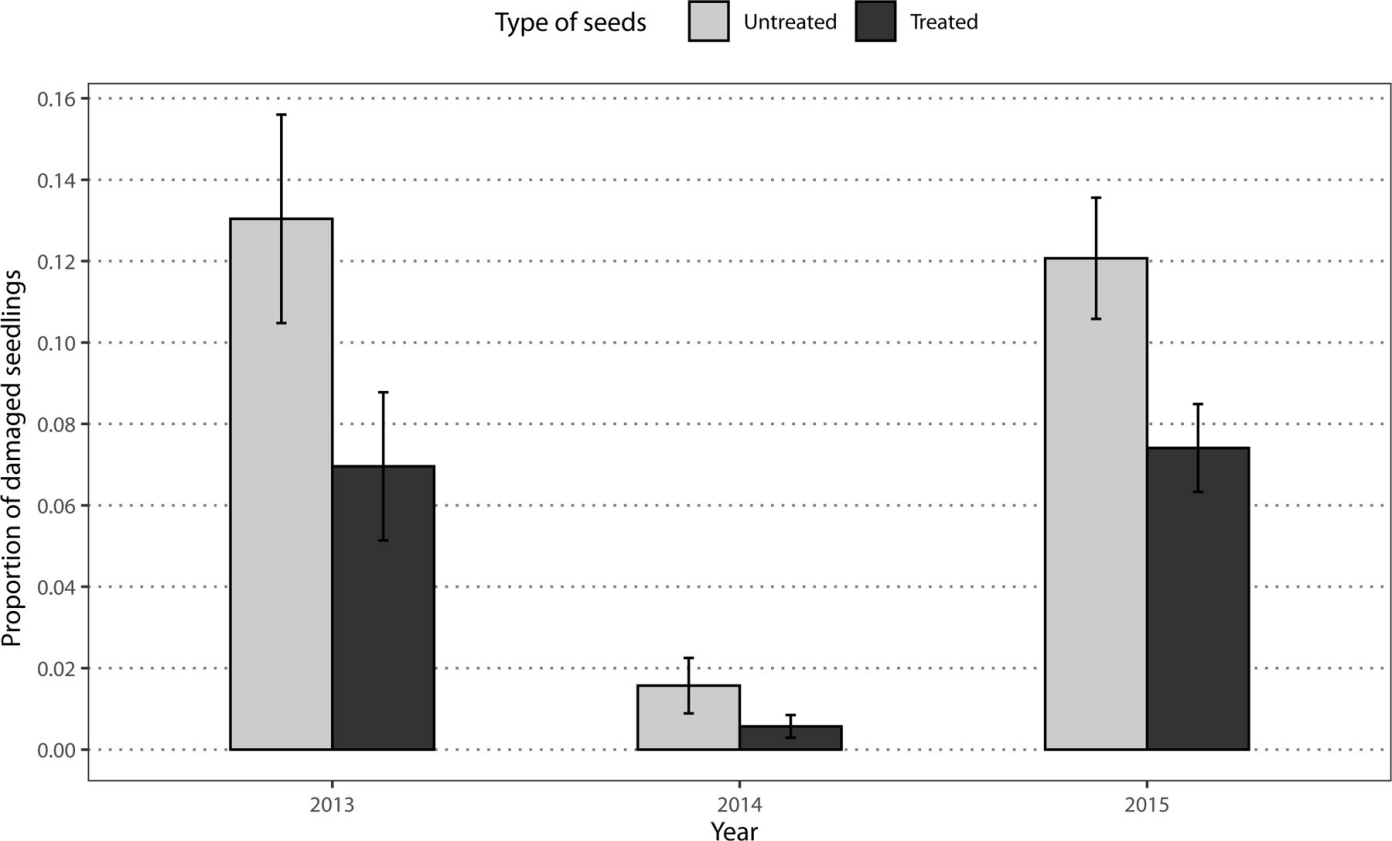
Seedling damage caused by soil-dwelling pests in 68 commercial corn fields in the province of Quebec, Canada, over a three-year period.

Visual inspection of three damaged plants in each plot showed that the damage associated with soil-dwelling insect pests in corn was characterized by a hole in the grain (caused by wireworms), or by smaller or less vigorous plants (caused indiscriminately by wireworms or seedcorn maggot larvae). Damage was observed in 8, 17 and 19 fields (62%, 89% and 79%) in 2013, 2014 and 2015, respectively. In all years, the percentage of corn seedlings damaged by soil-borne insect pests (wireworm, seedcorn maggot) was significantly higher at untreated stations (13.0%, 1.6% and 12.1% respectively) than in treated stations (7.0%, 0.6% and 7.4%) (LRT; χ^2^ = 8.11; df = 1; *p* = 0.004; Fig 4).

In soybean, very little damage to seedlings was observed, with only two sites presenting damage in 2015 (Nicolet: 1 plant/54 with a hole in the grain caused by wireworms; Roxton Pond: 3 plants/54 with stem damage caused by seedcorn maggot).

### Effects of neonicotinoids on yields

**Fig 5:**
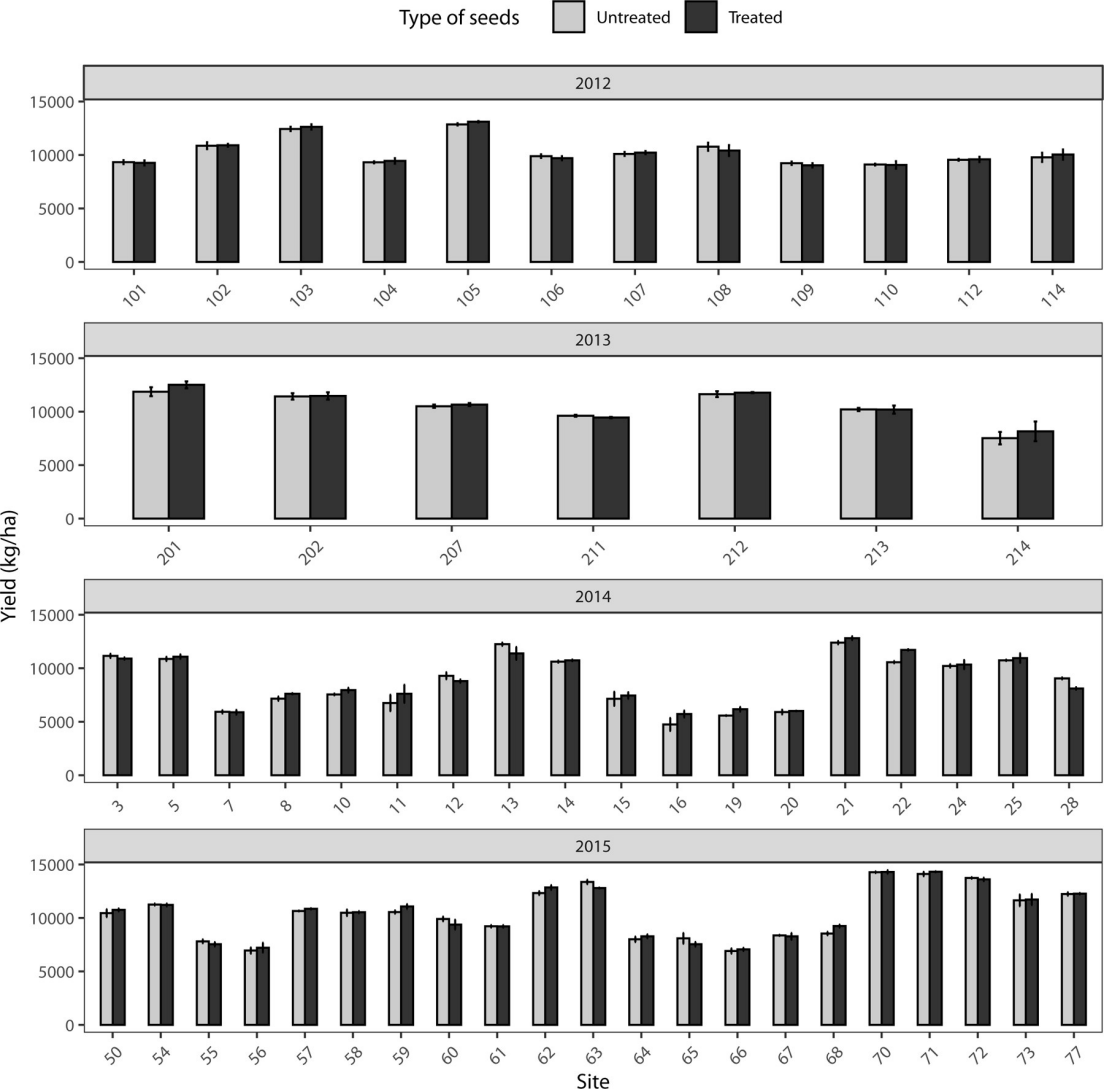
Mean yield (kg/ha) (±SE) in 68 commercial corn fields in the province of Quebec, Canada with neonicotinoid seed treatments or without (control strips).

In corn, yields were not significantly different between neonicotinoid treated and untreated strips regardless of the site, or the year (LRT; *F*_1, 186.42_ = 3.24; *p* = 0.073; Fig 5).

**Fig 6:**
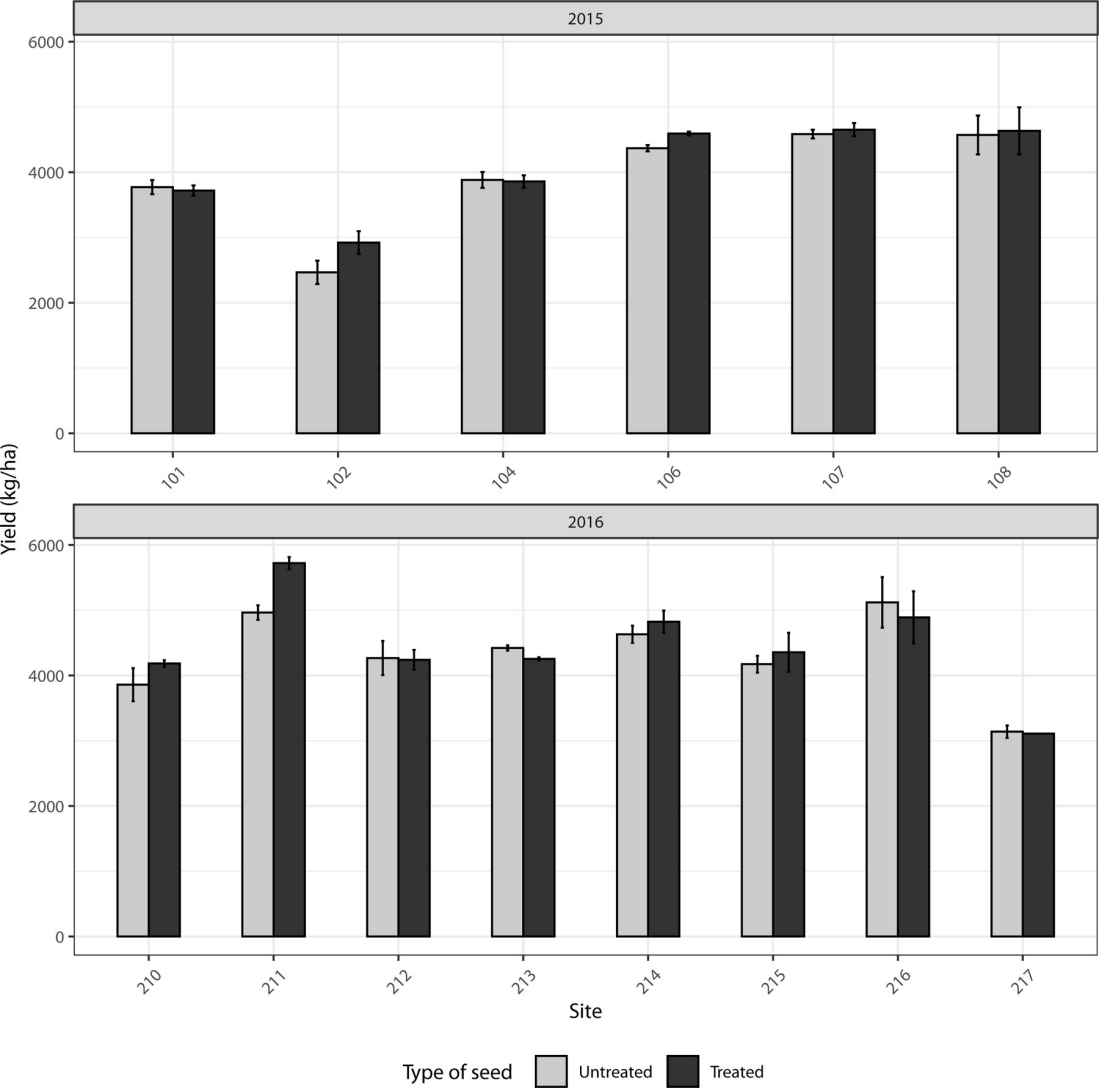
Mean yield (kg/ha) (±SE) in 15 commercial soybean fields in the province of Quebec, Canada with neonicotinoid seed treatments or without (control strips) in 2015 (A) and 2016 (B).

In soybean, yield did not differ significantly between neonicotinoid treated (4413 ± 170 kg/ha) and untreated strips (4330 ± 170 kg/ha) during the two-year study (LRT; *F*_1, 32.45_ = 1.54; *p* = 0.223) (Fig 6).

## Discussion

This study provides a strong demonstration that insecticide seed coatings on corn and soybean are not needed as a prophylactic control measure against soil-dwelling insect pests in field crops in Quebec, Canada. Overall, the abundance of such pests, including wireworms, was low in most fields monitored and no yield difference was observed between neonicotinoid seed treatments and control plots in corn or soybean.

### Effects of neonicotinoids on insect populations and damage

The main group of soil insect pests found at our corn study sites that could be managed by neonicotinoid seed treatments were wireworms. The seedcorn maggot was observed on only a few seedlings but did not cause major damage during the four-year study. White grubs were largely absent from our 68 sites. Corn rootworms were also monitored in 2012 and 2013 but were present in very low numbers [73]. This could be explained by the main rotation scheme used in Quebec (corn/soybean), which is known to reduce the prevalence of this pest species [74, 75]. Insect pressure was low at almost 90% of our corn study sites (below a threshold of 1 wireworm/bait trap), which is representative of the extensive surveys that have been done in the province since 2011 [36]. Therefore, IPM strategies for soil insect pests in Quebec corn fields should focus on wireworms.

In our study, with neonicotinoid seed treatments, no decrease in wireworm abundance was found from one year to another [36]. Some studies [76, 77] have shown that neonicotinoid treatments induce a prolonged intoxication of wireworms, making them moribund for several weeks, but do not reduce their populations. Our results confirm that those insecticides did not affect wireworm population levels.

The study by Furlan [78] showed that the type and intensity of damage to corn differed between wireworm species. In this study, the three species of *Agriotes* (*A*. *brevis*, *A*. *sordidus* and *A*. *ustulatus*) differed in length and did not affect corn in the same manner. In our case, the main wireworm species observed was *H*. *abbreviatus*; very little information is available on this species’ biology and food preferences and the damage it causes in corn. This species was first observed in organic soil in raspberry fields in Quebec [79, 80, 81, 82], but no information was available on the type and intensity of damage it causes in corn or soybean. We observed higher proportions of damaged corn seedlings in untreated corn plots in 8, 17 and 19 fields in 2013, 2014, and 2015, respectively. In Europe, the presence of 5 to 10 larvae per m^2^ caused 30% mortality of maize seedlings [83]. Furlan [78] observed that a loss of 1 corn plant per m^2^ could cause significant yield losses. In our study, we observed more than 10 larvae per m^2^ in 11 (58%) and 10 (42%) fields in 2014 and 2015, respectively, but no differences in plant stand or grain yield were observed. The main damage that was observed in the seedlings was a hole in the grain, which can cause growth delay and sometimes no growth at all. However, the consequences were generally limited to irregular plant heights in different portions of the field. This kind of damage, less intense than that caused by larger wireworm species such as *Agriotes* spp. [84] or *Melanotus* spp. [85, 86], could explain the lack of differences in corn plant stand or grain yield between treated and untreated strips.

These results call into question the threshold of 1 wireworm per bait trap [83] commonly used for our main wireworm species, *H*. *abbreviatus*. This threshold was developed for *Agriotes* spp. found in northern France. Furlan [78] demonstrated that thresholds could differ with wireworm species and the type of damage they are causing. He identified thresholds varying between 1 and 5 wireworms per bait trap for species with a length between 40 mm and 12 mm. In our case, *H*. *abbreviatus* measured 12 mm at the last larval stage [36] and did not cause significant damage to corn plants. A study conducted in 2016 at 162 sites in Quebec showed that 5% seedling damage was observed when more than three wireworms/bait traps were found in the fields [87]. This suggests that the threshold for *H*. *abbreviatus* could be closer to three wireworms per bait trap, but this remains to be validated [87]. This low pest pressure, due in part to the prevalence of *H*. *abbreviatus*, a different wireworm species than the ones observed in other parts of Canada or the USA [88, 89, 90], could explain the lack of yield differences in our study.

In soybean, although high wireworm populations were observed in some fields, almost no damaged grains or plants were observed. Wireworms feed mainly on cereals [37, 84]; hence, soybean, a legume species, may not be an adequate food source for larvae. Although other soil insect pests could pose a threat to soybean, such as white grubs or seedcorn maggot, very small populations of those pests have been observed in monitored fields. However, they could become a problem in some years, sporadically, when harsh weather conditions are experienced [47, 48, 91, 92]. The wet springs that sometimes occur in Quebec can increase the damage caused by those pests, and insecticide seed treatments could be useful in such conditions. Soybean aphid, *Aphis glycines* (Matsumura), is one of the pest species targeted by neonicotinoid seed treatments. In one study [93], observations of soybean aphid populations showed that the threshold was not reached in all fields in either year. Aphids emerged too late in fields to be controlled by insecticide seed treatments, a finding also reported in other studies in the United States and Quebec [93, 94]. Overall, very low pressure associated with soil insect pests were observed in soybean in both years of the study.

### Effects of neonicotinoids on corn and soybean yields

Overall, our study did not show any differences in grain yield between treated and untreated corn or soybean seed. Several factors may help explain these results, such as low pest pressure, compensatory growth, rapid decrease in neonicotinoid concentrations within the plants, or the absence of other non-abiotic stresses.

Some studies have reported an increase in yield associated with insecticide seed treatment when wireworms were present in high abundance [61] or when more than two pest species were present [51]. For example, Wilde et al. [61] evaluated the effect of seed treatments on wireworms in corn and found that this approach increased plant stands and grain yield almost 50% of the time, mainly when insects were present in high numbers. The same conclusion emerged from an overall analysis of soybean yield increases across the USA [51, 56], in which insecticide seed treatment was found to be useful only when three foliar-feeding pest species were present at the beginning of the summer. Cox and Cherney [48] showed, however, that there is high variability in soybean yield for the same varieties and seed densities at different locations in North America, and that the use of insecticide seed treatments did not provide benefits to all growers. In corn, a meta-analysis combining 15 years of U.S. data revealed that even at the highest dose used against corn rootworm, there was no significant benefit of using seed treated corn [30]. However, North et al. [59] found the opposite results in an analysis of 91 trials on 14 years in mid-south USA, with global yield gain of 700 kg/ha in corn treated with neonicotinoids. Such a high level of variability between studies could be explained by climatic conditions, which varied between sites and years, by the abundance of pest species and by the efficiency of the insecticides. Alford and Krupke [95] reported that less than 1.5% of clothianidin applied to the seeds translocate through the roots and shoots of corn plants under field conditions and that this treatment did not cover the entire window of activity of all soil insect pests. This temporally limited protection from insecticide within the plant could in part explain the variability in yield differences between treated and untreated plants in many field studies [57, 58, 61].

Overall, insect pest pressure was low in the five years of the study. Wireworm populations were below the threshold of 1 wireworm/bait trap in 69% of the soybean fields and in 92% of the corn fields. If we consider a threshold of 3 wireworms/bait traps, the threshold was reached in only 2 corn fields. White grub and seedcorn maggot numbers were also very low in all our fields. Even though some damage to seedlings was observed in corn fields and plant stands were greater in treated soybean plots, no overall differences in yield were observed.

A "stress shield" or growth facilitation effect has been observed with neonicotinoid treated seedlings in a few studies on corn, sorghum and wheat [96, 97, 98, 99]. Increased growth has been observed for neonicotinoid treated seeds compared to untreated seeds when exposed to different stresses [96, 97, 98, 99]. This "stress shield" may be observed mainly in response to drought stress or weed pressure. In our study, however, no such effect has been observed, which could be explained by compensatory growth. Compensatory growth is the increase that occurs in plant growth rate following a period of stress, such as drought, or increased plant population density [100, 101]. This compensatory growth is well documented in corn [30, 96, 100, 101, 102] and soybean [52, 103]. The stress caused by soil insect pests feeding on young seedlings could have triggered this phenomenon of compensatory growth, which would explain the lack of difference in yield.

Various IPM strategies have been developed in recent years for soil insect pests with the aim of reducing the use of insecticide seed treatment. Pest management of wireworms does not require the prophylactic use of neonicotinoids and in cases where pest densities are high, alternatives to insecticides exist. Some approaches are still being tested, such as mass trapping of adult wireworms with light traps [104], crop rotations with brown mustard or buckwheat [105], attraction to insecticide-treated wheat grown between untreated potato rows [106]; they represent alternative control measures that are under development in Canada. Other methods, such as trap crops using pea and lentil [107] or the use of entomopathogenic fungi such as *Metarhizium anisopliae* [108, 109, 110] could be tested on a large scale against wireworms. Furlan et al. [111] proposed a mutual funds approach covering the risk of implementing IPM programs for Italian producers, which increased farmer profits while reducing the use of pesticides. In Quebec, a decision support tool was developed based on a boosted regression analysis of all physical and landscape parameters that favour the presence of the main wireworm species in the province, *H*. *abbreviatus*. This tool is freely available online (VFF Qc, available at www.cerom.qc.ca/vffqc), and allows producers to predict the risk of encountering a high abundance of this wireworm species [87].

Our study clearly demonstrates that neonicotinoid seed treatments in corn and soybean are not justified in about 95% of the field crop acreage in the province of Quebec, which represents 500,000 ha of fields (corn, soybean and cereals). While the use of neonicotinoids is to be phased out completely in Canada by 2021 [112], other insecticide treatments are replacing them; they are based on the same marketing strategy of insurance against the risk of pest attack. With these new products, the same limited availability of untreated seed is observed as is the case for neonicotinoids. The widespread use of insecticides as seed treatments—even if the new products are potentially less harmful to the environment and human health—will not be sustainable over the long term, and will increase risk of the insects developing resistance [2, 30] along with contamination of the environment. An exponential increase has been observed in the levels of these new insecticides in rivers in Quebec since they were registered as seed treatments; they are already reaching the maximum allowable concentrations for aquatic life in some places [17]. IPM strategies based on pest densities and risk factors represent a more sustainable solution for protecting field crop from threats and for preserving the environment and human health.

## Supporting information

S1 Table Characteristics of the 84 experimental sites followed between 2012 and 2016 in corn and soybean in Quebec, Canada. Fertilization: Organic fertilization involves the use of manure, but unspecified.
(CSV)
